# The Relationship between Pre-Pandemic Interferon Gamma Release Assay Test Results and COVID-19 Infection: Potential Prognostic Value of Indeterminate IFN-*γ* Release Assay Results

**DOI:** 10.1155/2021/1989277

**Published:** 2021-07-31

**Authors:** Sermin Borekci, Fatma Gulsum Karakas, Serhat Sirekbasan, Bahar Kubat, Rıdvan Karaali, Gunay Can, Bekir Sami Kocazeybek, Bilun Gemicioglu

**Affiliations:** ^1^Department of Pulmonary Diseases, Cerrahpasa Medical Faculty, Istanbul University-Cerrahpasa, Istanbul, Turkey; ^2^Department of Medical Laboratory Techniques, Eldivan Vocational School of Health Services, Cankırı Karatekin University, Cankırı, Turkey; ^3^Department of Infectious Diseases, Cerrahpasa Medical Faculty, Istanbul University-Cerrahpasa, Istanbul, Turkey; ^4^Department of Public Health, Cerrahpasa Medical Faculty, Istanbul University-Cerrahpasa, Istanbul, Turkey; ^5^Department of Medical Microbiology, Cerrahpasa Medical Faculty, Istanbul University-Cerrahpasa, Istanbul, Turkey

## Abstract

**Objective:**

To reveal the relationship between interferon-gamma release assay (IGRA) test (Standard ETB-Feron ELISA (TBF)) results performed within 12 months before the COVID-19 pandemic and the frequency of COVID-19 infections and the severity of COVID-19.

**Methods:**

The retrospective TBF test results and contact information of 684 patients aged over 18 years who underwent TBF testing between March 11th, 2019, and March 10th, 2020, were obtained. Of the 684 patients contacted by phone, 365 agreed to participate in the study and were enrolled. The patients were divided into three groups (TBF test positive, negative, and indeterminate). The data obtained from the questionnaire were compared statistically.

**Results:**

According to the TBF test results, positive (*n* = 51, 14%), negative (*n* = 286, 78.3%), and indeterminate (*n* = 28, 7.7%) groups were compared. The frequency of COVID-19 infections in the indeterminate group was found significantly higher than that in the positive and negative groups (*p*=0.005). When the group with COVID-19 (*n* = 46, 12.6%) was compared with the group without (*n* = 319, 87.4%), no difference was found in terms of age, sex, body mass index, smoking history and number of cigarettes smoked, TB history, diabetes mellitus, hypertension, coronary artery disease, and biologic and corticosteroid therapy use. Only the frequency of obstructive pulmonary disease was significantly higher in the group without COVID-19 (*p*=0.033).

**Conclusion:**

The frequency of COVID-19 infection was increased in patients with indeterminate TBF test results. Indeterminate TBF test results may be a guide in terms of risk stratification in groups at risk for COVID-19.

## 1. Introduction

Coronavirus disease 2019 (COVID-19), an infectious disease caused by the severe acute respiratory syndrome coronavirus 2 (SARS-CoV-2) virus, affected the whole world after the first case was reported in Wuhan, China, in December 2019 [1]. While the COVID-19 pandemic continues its impact with high mortality and morbidity, the search to reduce these effects continues.

As seen before with other coronaviruses (SARS-CoV and MERS-CoV), severe immune dysregulation is also responsible for response to the SARS-CoV-2 virus. The excessive response of the innate immunity and the accompanying reduction of lymphocyte subsets (CD4^+^ T cells, CD8^+^ T cells, CD19^+^ B cells, and natural killer cells) causes widespread damage in all organs, especially the pulmonary parenchyma. However, it produces a systemic hyperinflammatory and hypercoagulative state [2]. In one study, SARS-CoV-2-specific CD8^+^ and CD4^+^ T cells were identified in 70% and 100% of patients recovering from COVID-19, respectively [3]. In another study, it was determined that T cells specific to SARS-CoV-2 predominantly produced effector and T helper 1 (Th1) cytokines, and additionally, Th2 and Th17 cytokines were found. It has been shown that SARS-CoV-2-specific T cells exist relatively early and increase over time. It has been interpreted that disease severity and variability in T cell responses may be related to each other [4]. After being infected by virus, the B cells are assisted by T cells and differentiate into plasma cells and produce antibodies specific to a viral antigen. A neutralizing natural antibody is efficient at blocking the virus from entering into host cells to limit the infection and has an important role at the later stage of infection and prevents relapse of infection [5]. On the other hand, T lymphocytes mediate a cellular immunity response inside the infected cells. The whole adaptive immune response is directed by helper T cells, and cytotoxic T cells play a vital role in the clearance and cleaning of viral-infected cells [6]. The data obtained show that immunopathology, including the T cell response, plays a crucial role in COVID-19 [7].

Interferon-gamma (IFN-*γ*) is an important cytokine that is primarily produced by cells of the immune system, including innate-like lymphocytes, such as natural killer cells and innate lymphoid cells, and adaptive immune cells, such as Th1 cells and CD8^+^ cytotoxic T lymphocytes. Signals generated by the IFN-*γ* receptor activate the Janus kinase (JAK)-signal transducer and activator of transcription 1 (STAT1) pathway to induce expression of several genes that have necessary immune effector functions. IFN-*γ* also has critical roles in regulating the functions of specialized tissue cells, has effects on progenitor and stem cells, and participates in tissue and organ function under homeostatic, immune, and pathological conditions [8].

Interferon-gamma release assay (IGRA) tests are in vitro tests that measure the cellular immune response against *Mycobacterium tuberculosis* antigens [9, 10]. In these tests, the IFN-*γ* response released from the cells reminiscent of the antigen is measured when previously sensitized memory T cells are restimulated with specific antigens. Although its primary purpose is to detect latent tuberculosis infection (LTBI), it is also crucial in showing memory T cell functions. A study conducted in Italy showed that IGRA tests could be used to predict mortality in patients with severe COVID-19 [2]. Approximately 2–11% of IGRA tests result as “indeterminate.” Patients with an indeterminate response are those with chronic disease or whose immunity is compromised, in which an adequate immune response is not formed against a mitogen control [2, 11]. Also, low lymphocyte counts may cause false-negative or indeterminate results due to the insufficient immune response [12, 13].

The Standard E TB-Feron enzyme-linked immunosorbent assay (ELISA) (TBF; SD Biosensor Inc., Gyeonggi-do, Republic of Korea) test is also one of the interferon-gamma release tests. This test is based on the determination of the IFN-*γ* levels secreted by T cells stimulated with specific antigens using the ELISA method [14, 15].

Hence, our study's aim is to reveal the relationship between TBF test results performed within 12 months before the COVID-19 pandemic and the frequency of COVID-19 infections and the severity of COVID-19 based on the hypothesis that TBF test results might be an indirect indicator of T cell functions via T memory cells.

## 2. Materials and Methods

### 2.1. Study Setting and Participants

The study was planned as a retrospective cross-sectional descriptive study. Patients with TBF tested by our University's Medical Microbiology Department until 12 months before the first COVID-19 cases in Turkey were enrolled in the study. Thus, the status of T cell functions was tried to be represented using T memory cells before the pandemic. The TBF test results and contact information of 684 patients aged over 18 years were obtained from the hospital automation system. Of these 684 patients, those who consented to participate in the study and who had a perception level to understand and answer the questionnaire questions were enrolled. The questionnaire included questions about age, sex, height, weight, smoking history, whether they have had a COVID-19 infection, the severity if they had, the COVID-19 treatment received at that time, accompanying diseases, drugs they regularly used, and whether they had received a COVID-19 vaccine. According to the TBF test results, the patients were divided into three groups: positive, negative, and indeterminate, and the data obtained were compared statistically.

### 2.2. Standard E TB-Feron ELISA (TBF) Test

A TBF test measures the level of antigen-specific IFN-*γ* released from T-lymphocytes using the ELISA method in whole blood from susceptible individuals. TBF test measures IFN-γ after stimulation of heparinized whole blood cells with *M. tuberculosis* specific antigens (ESAT-6 (Early Secretory Antigenic Target 6), CFP-10 (Culture Filtrate Protein 10), and TB-7.7 (Tuberculosis antigen 7.7)). The method is based on the determination of IFN-*γ* release under in vitro conditions using the ELISA method. Blood sent from various polyclinics and wards of our hospital was studied according to the manufacturer's instructions (SD Biosensor Inc., Gyeonggi-do, Republic of Korea) using the ELISA method, which measures *M. tuberculosis* proteins and IFN-*γ* levels released by sensitive T lymphocytes [15].

For the application of the TBF test, blood was taken into nil tubes, TB-specific antigen (ESAT 6, CFP 10, TB7.7 as antigen), and mitogen tubes for the negative control. The mitogen tube was used as a positive control in the TBF test. After taking 1 mL of blood into each tube, it was mixed by inverting several times to cover the inner surfaces of the tubes entirely with blood. In the TBF test, blood samples taken from the patient were placed in these special tubes and incubated for 16 to 24 hours, and the presence of IFN-*γ* produced in response to specific peptide antigens was investigated. After the incubation, the tubes were centrifuged to separate plasma, and the amount of IFN-*γ* (IU/mL) was measured using ELISA.

### 2.3. Evaluation of TBF Test

The evaluation starts when the difference between the mitogen and nil tubes is 0.5. If the value is less than 0.5, the patient's immune response is considered to be insufficient, and the result is reported as “indeterminate.” After the difference between the mitogen and nil tubes is found as 0.5, the difference between the TB antigen and nil tube is calculated; if the difference is ≥0.35, the test is positive; if <0.35, the test is negative. Also, high IFN-*γ* response to the nil control or insufficient response to mitogen is considered as an indeterminate result. The interpretation of TBF is specified in Table 1.

### 2.4. Anthropometric Data

Patients' height (cm), weight (kg), and body mass index (BMI) (kg/m^2^) were recorded. According to the BMI value, they were classified as underweight (<18.5 kg/m^2^), normal (18.5–24.9 kg/m^2^), overweight (25–29.9 kg/m^2^), and obese (>30 kg/m^2^) [16].

### 2.5. Disease Severity

This was classified as outpatient, hospital treatment, and intensive care treatment.

### 2.6. Statistical Analysis

Statistical analyses were performed using statistical software (IBM SPSS, version 20.0; SPSS Inc., Chicago, IL, USA). Categorical variables are expressed as a percentage. The chi-square and Fisher exact tests were used to compare the groups. The significance level was set at *p* < 0.05.

### 2.7. Ethics Statement

All procedures performed in studies involving human participants were in accordance with the ethical standards of the institutional and/or national research committee (IRB No. 604.01.1-34109) and were conducted according to the 1964 Helsinki Declaration and its later amendments or comparable ethical standards. Verbal informed consent was obtained from all individual participants included in the study.

## 3. Results

**Figure 1:**
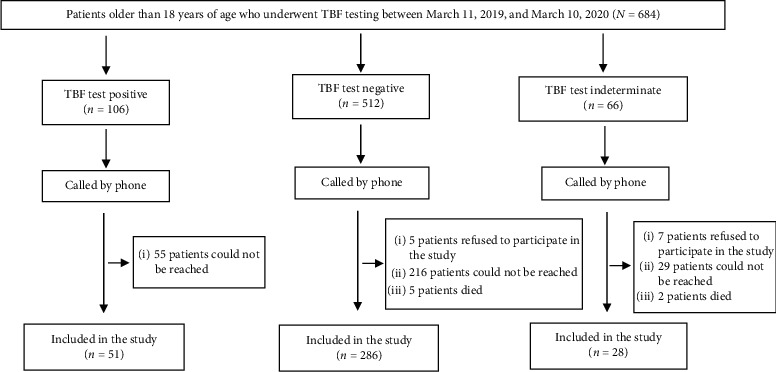
Flow chart.

Among the 684 patients over the age of 18 years who were tested for TBF between March 11th, 2019, and March 11th, 2020, 365 patients who met the inclusion criteria were included in the study. The follow-up scheme of the patients is shown in Figure 1.

One hundred seventy-five of the patients were male, 190 were female, and their mean age was 42.6 ± 16.7 (range, 18–92) years. The demographic data of the patients are shown in Table 2.

None of the patients was receiving dexamethasone treatment above a dose of 10 mg/day, and none who had a COVID-19 infection received treatment in intensive care.

The groups with positive (*n* = 51, 14%), negative (*n* = 286, 78.3%), and indeterminate (*n* = 28, 7.7%) results were compared, and the frequency of having COVID-19 was found to be significantly higher in the indeterminate group than in the positive and negative groups (*p*=0.005). Male sex, advanced age, and average cigarette consumption over 20 pack-years were significantly higher in the group with positive TBF results than in the other groups (*p*=0.001, *p* ≤ 0.001, and *p*=0.003, respectively). In the group with indeterminate TBF tests, the presence of CRF was significantly higher than that in the other groups (*p*=0.001). In the groups with positive and indeterminate TBF tests, TB frequency (*p* ≤ 0.001) and the presence of hypertension (*p*=0.010) were found to be significantly higher compared with the group with the negative TBF test results.  Table 3 shows the parameters by which the groups were compared according to the TBF test results.

Four (14.2%) of 28 patients with indeterminate TBF results had lymphopenia (<1.0 × 10^9^/L) in blood counts simultaneously with the TBF test.

The frequency of obstructive pulmonary disease was found to be higher in the group with COVID-19 (*n* = 46, 12.6%) than the group without COVID-19 (*n* = 319, 87.4%) (*p*=0.033).  Table 4 shows the parameters compared in the groups with and without COVID-19.

## 4. Discussion

In this study, based on the hypothesis that the TBF test, which is one of the IGRA tests, might be an indirect indicator of T cell functions through T memory cells, the frequency of COVID-19 infections was found to be significantly higher in the group with indeterminate TBF test results. Indeterminate TBF test results may help identify priority risk in groups at risk for COVID-19. No relationship was found between the frequency of COVID-19 and age, sex, the presence of comorbidity, smoking, and corticosteroid and biologic therapy use.

Two studies have investigated the relationship between IGRA test results and COVID-19 disease [2, 17]. In the study of Torre et al. [2], which included 335 patients with severe COVID-19 who received immunosuppressive therapy and underwent IGRA tests, the death rate in patients with indeterminate IGRA results was significantly higher than that in the group with positive and negative results (*p*=0.38). In the study of Solanich et al. [17], which included 96 patients hospitalized for COVID-19 infection and who underwent IGRA tests (QuantiFERON-TB Gold Plus), indeterminate IGRA test results were found to be significantly higher in patients with severe COVID-19. Our study found that the frequency of COVID-19 infection was significantly higher in patients with indeterminate test results according to the TBF test performed before COVID-19 infection. Torre et al. and Solanich et al. recruited hospitalized COVID-19 patients in their studies. It is not surprising to find indeterminate IGRA test results, especially in severe cases, because of the severe immune dysregulation and decreased lymphocyte subsets in COVID-19 infections. Our study included patients who had an IGRA test before COVID-19. In this respect, it is different from the other two studies. In our study, the finding that indeterminate IGRA test results before COVID-19 were associated with increased COVID-19 frequency might be a guide in determining more risky groups in daily practice. In societies where the entire population cannot be vaccinated during the pandemic period, this result may help determine the priority groups for vaccination. In our study, the low vaccination ratio of the three groups and the finding of no difference regarding vaccination ratios between the groups support this assertion.

The IGRA test also studied in HIV patients in recent years and reported that HIV-1-infected patients with an indeterminate IFN-*γ* release assay result at baseline were at significantly higher risk of developing AIDS manifestations other than tuberculosis regardless of CD4^+^ T cell count [18]. This result supports our findings that “patients with indeterminate IGRA test results are in more risky groups and clinicians should be careful in this regard.”

Although data regarding the relationship between IGRA test results obtained after stimulation with tuberculosis-specific antigens and COVID-19 infection are limited in the literature, it brought to mind the question of whether an IGRA test designed with SARS-CoV-2-specific antigens could be used to detect COVID-19-specific cellular immunity. Studies on IGRA tests specific to SARS-CoV-2 have been completed rapidly, and data on the first SARS-CoV-2-specific IGRA tests have been published very recently [19, 20]. Time will show the benefits of these tests in diagnosing the disease and in the management of the pandemic.

With the prediction that the COVID-19 pandemic is milder in countries where LTBI is common, it has been hypothesized that the immunity provided with LTBI may have a protective effect against COVID-19 [21]. Few studies are investigating the relationship between LTBI and COVID-19 with this hypothesis. In the study of Takahashi [1], it was shown that LTBI was associated with a decrease in the COVID-19 mortality rate. In our study, no difference was found between the TBF test-positive group and the TBF test-negative group regarding the frequency of COVID-19. It is noteworthy that there was no difference in the frequency of COVID-19. However, the frequency of TB was significantly higher in the TBF-positive group than the negative group. In our study, when the severity of the disease was divided into three groups as “outpatient, hospital, and intensive care treatment,” no difference was found between both TBF outcome groups and groups with and without COVID-19. However, the fact that no patient was admitted to intensive care in our study may be because some of the patients admitted to intensive care died. Nevertheless, the rates of outpatient and inpatient treatment were similar when the groups were compared, suggesting that the severity of the disease in patients with LTBI (TBF-positive group) and those without LTBI (TBF negative) did not differ. These findings are different from the result of Takahashi and do not support the hypothesis that immunity provided by LTBI may have a protective effect against COVID-19. Studies with more extensive series are needed on this subject.

When looking at the relationship between COVID-19 and chronic disease, studies report that diabetes mellitus, chronic obstructive pulmonary disease (COPD), obesity, and cardiovascular diseases affect the progression and symptoms of COVID-19 [21, 22]. In our study, no difference was found between the groups with and without COVID-19 in terms of hypertension, diabetes mellitus, coronary artery disease, chronic renal failure, and the presence of malignancy. In contrast, the frequency of obstructive pulmonary disease was significantly lower in the group with COVID-19. This may be because people with obstructive pulmonary disease are more aware of the severity of COVID-19 and use their medications better and take better personal protective measures. As a matter of fact, in this study, most of the patients who had TBF tests were followed up in neurology, gastroenterology, rheumatology, and dermatology departments in a university hospital. Therefore, their awareness of infection protection measures may have increased. The relationship between treatments such as inhaled steroids and Beta 2 agonists used in COPD and COVID-19 is still a pending research subject [23]. In our study, a comparison could not be made because the details of the treatments of inhaled inhabitants used by the patients were not questioned.

It has been shown that smoking facilitates SARS-CoV-2 infection by increasing angiotensin-converting enzyme (ACE)-2 receptor synthesis and causes severe symptoms and severe illness [24, 25]. By contrast, some studies reported that the frequency of COVID-19 did not increase in smokers [26]. In our study, no difference was found between the group with and without COVID-19 regarding smoking history and the number of cigarettes smoked.

Corticosteroids and other immunosuppressive drugs are a cause of indeterminate IGRA test results [17]. In our study, no difference was found between the positive, negative, and indeterminate TBF test groups regarding the frequency of corticosteroid and biologic therapy use. Besides, similar results were found between groups with and without COVID-19 regarding the frequency of corticosteroid and biologic therapy use. This may be because most patients received low-dose (5–10 mg/day dexamethasone) steroid therapy, with doses not high enough to cause immunosuppression.

The limitation of our study is that the data except the TBF test were based on the verbal statements of the patients. However, its strength is that it is the first study to investigate the frequency and severity of COVID-19 with IGRA tests performed before the pandemic.

## 5. Conclusions

As a result, patients with indeterminate TBF test results were found to have an increased frequency of COVID-19 infection. Indeterminate TBF test results may be a guide in terms of risk stratification in groups at risk for COVID-19. Studies with more extensive series are needed on this subject.

## Figures and Tables

**Table 1:** Evaluation of TBF test.

Results	Mitogen-nil value	TB-specific antigen-nil value (IU/mL)
TBF positive	≥0.5	≥0.35
TBF negative	≥0.5	<0.35
Indeterminate	<0.5	<0.35 vs nil ≤8.0

**Table 2:** Demographic characteristics of the patients.

Parameters	
Female/male, *n*	190/175
Age (mean ± SD)	42.6 ± 16.7

Age (year), *n* (%)	
<30	97 (36.6)
31–50	160 (43.8)
>50	108 (29.6)

Body mass index, (kg/m^2^), *n* (%)	
Underweight (<18.5)	49 (13.4)
Normal weight (18.5–24.9)	132 (36.2)
Overweight (24.5–29.9)	104 (28.5)
Obese (>30)	80 (21.9)

Smoking	
Never smoker, *n* (%)	205 (56.2)
Former smoker, *n* (%)	61 (16.7)
Current smoker, *n* (%)	99 (27.1)

Smoking amount (pack-year), *n* (%)	
0	205 (56.2)
1–10	60 (16.4)
11–20	58 (15.9)
>20	42 (11.5)
TB pass, *n* (%)	12 (3.3)

Subgroup analysis of TB patients (*n* = 12), *n* (%)	
Lung TB	5 (41.7)
Extrapulmonary TB	4 (33.3)
Unknown	3 (25.0)

TBF result, *n* (%)	
Positive	51 (14.0)
Negative	286 (78.3)
Indeterminate	28 (7.7)
TBF prompting unit, *n* (%)	52 (14.2)
Neurology	30 (8.2)
Gastroenterology	232 (63.6)
Rheumatology	9 (2.5)
Dermatology	42 (11.5)
Other	52 (14.2)

Chronic disease, *n* (%)	
HT	40 (11.0)
DM	32 (8.8)
Coronary artery disease	23 (6.3)
Rheumatologic disease	174 (47.7)
Obstructive lung diseases	54 (14.8)
MS	17 (4.7)
Malignancy	15 (4.1)
Hypothyroidism	16 (4.4)
Cardiac arrhythmia	7 (1.9)
IBD	35 (9.6)
CRF	11 (3.0)

Drugs used, *n* (%)	
Biological treatments	169 (46.3)
Corticosteroid	130 (35.6)

COVID-19 status, *n* (%)	
Passed (PCR positive)	46 (11.5)

Severity of patients with COVID-19, *n* (%)	
Outpatient treatment	32 (69.6)
Treatment in the hospital	14 (30.4)

Total length of hospital stay, *n* (%)	
1–5 days	5 (35.7)
6–10 days	6 (42.9)
>10 days	3 (21.4)

Treatment of 46 patients with COVID-19, *n* (%)	
Follow-up without treatment	11 (23.9)
Favipiravir	23 (50.0)
Plaquenil	6 (13.0)
Favipiravir + plaquenil	5 (10.9)
Anticoagulant	9 (19.5)
Antibiotic	2 (4.2)
Oxygen support	1 (2.2)
Unknown	1 (2.2)

COVID-19 vaccine status, *n* (%)	
Not vaccinated	315 (86.3)
Vaccinated	50 (13.7)

Reason for not being vaccinated, *n* (%)	
Due to the age limit for indication	304 (96.5)
He/she refused to be vaccinated	11 (3.5)

TBF: Standard E TB-Feron ELISA (TBF) test; HT: hypertension; DM: diabetes mellitus; MS: multiple sclerosis; IBD: inflammatory bowel disease; CRF: chronic renal failure.

**Table 3:** Comparison of groups according to TBF test results.

Parameters	TBF positive (*n* = 51)	TBF negative (*n* = 286)	TBF indeterminate (*n* = 28)	*p*
Sex, *n* (%)				
Male	**36 (70.6)**	123 (43.0)	16 (57.1)	**0.001** ^*∗*^
Female	15 (29.4)	163 (57.0)	12 (42.9)	

Age (year), *n* (%)				
<30	3 (5.9)	87 (30.4)	7 (25.0)	**≤0.001** ^*∗*^
31–50	18 (35.3)	131 (45.8)	11 (39.3)	
>50	**30 (58.8)**	68 (23.8)	10 (35.7)	

Body mass index, (kg/m^2^), *n* (%)				
Underweight (<18.5 kg/m^2^)	2 (3.9)	41 (14.3)	6 (21.4)	0.124
Normal weight (18.5–24.9 kg/m^2^)	15 (29.4)	109 (38.1)	8 (28.6)	
Overweight (24.5–29.9 kg/m^2^)	20 (39.2)	75 (26.3)	9 (32.1)	
Obese (>30 kg/m^2^)	14 (27.5)	61 (21.3)	5 (17.9)	

Smoking				
Never smoker, *n* (%)	26 (51.0)	166 (58.0)	13 (46.4)	0.124
Former smoker, *n* (%)	13 (25.5)	40 (14.0)	8 (28.6)	
Current smoker, *n* (%)	12 (23.5)	80 (28.0)	7 (25.0)	

Smoking amount (pack-year), *n* (%)				
0	26 (51.0)	166 (58.0)	13 (46.4)	0.003^*∗*^
1–10	2 (3.9)	54 (18.9)	4 (14.3)	
11–20	10 (19.6)	42 (14.7)	6 (21.4)	
>20	**13 (25.5)**	24 (18.4)	5 (17.9)	

TB pass, *n* (%)				
Yes	**6 (11.8)**	3 (1.0)	**3 (10.7)**	≤0.001^*∗*^
No	45 (88.2)	283 (99.0)	25 (89.3)	

TB subgroup, *n* (%)				
Lung TB	3 (50.0)	0 (00.0)	2 (66.7)	0.138
Extrapulmonary TB	3 (50.0)	1 (33.3)	0 (00.0)	
Unknown	0 (00.0)	2 (66.7)	1 (33.3)	

Chronic disease *n* (%)				
HT	**11 (21.6)**	24 (8.4)	**5 (17.9)**	**0.010** ^*∗*^
DM	4 (7.8)	23 (8.0)	5 (17.9)	0.209
Rheumatologic disease	27 (52.9)	137 (47.9)	10 (35.7)	0.336
Coronary artery disease	4 (7.8)	18 (6.3)	1 (3.6)	0.756
Obstructive lung diseases	4 (7.8)	18 (6.3)	1 (3.6)	0.231
MS	2 (3.9)	12 (4.2)	3 (10.7)	0.285
Malignancy	1 (2.0)	11 (3.8)	3 (0.7)	0.154
Hypothyroidism	2 (3.9)	12 (4.2)	2 (7.1)	0.756
Cardiac arrhythmia	1 (2.0)	5 (1.7)	1 (3.6)	0.798
IBD	6 (11.8)	26 (9.1)	3 (10.7)	0.818
CRF	2 (3.9)	5 (1.7)	**4 (14.3)**	**0.001** ^*∗*^

Drugs used, *n* (%)				
Biological treatments	23 (45.1)	137 (47.9)	9 (32.1)	0.275
Corticosteroid	16 (31.4)	105 (36.7)	9 (32.1)	0.705

COVID-19 status, *n* (%)				
Passed (PCR positive)	6 (11.8)	31 (10.8)	**9 (32.1)**	**0.005** ^*∗*^

Weight of patients with COVID-19, *n* (%)				
Outpatient treatment	4 (66.7)	22 (71.0)	6 (66.7)	0.950
Treatment in the hospital	2 (33.3)	9 (29.0)	3 (33.3)	
Intensive care treatment	0 (00.0)	0 (00.0)	0 (00.0)	

COVID-19 vaccine status, *n* (%)				
Done	10 (19.6)	37 (12.9)	3 (10.7)	0.395
Not done	41 (80.4)	249 (87.1)	25 (89.3)	

CRF: chronic renal failure; DM: diabetes mellitus; HT: hypertension; IBD: inflammatory bowel disease; MS: multiple sclerosis; TBF: Standard ETB-Feron ELISA (TBF) test. ^*∗*^*p* value < 0.05 was considered statistically significant. Values below 0.05 are in bold and indicate statistically significant differences between two categories.

**Table 4:** Comparison of groups with and without COVID-19.

Parameters	COVID-19 (+) (*n* = 46)	COVID-19 (−) (*n* = 319)	*p*
Sex, *n* (%)			
Male	27 (58.7)	148 (46.4)	0.118
Female	19 (41.3)	171 (53.60)	

Age (year), *n* (%)			
<30	9 (19.6)	88 (27.6)	0.284
31–50	25 (54.3)	135 (42.3)	
>50	12 (26.1)	96 (30.1)	

Body mass index (kg/m^2^), *n* (%)			
Underweight (<18.5 kg/m^2^)	4 (8.7)	45 (14.1)	0.489
Normal weight (18.5–24.9 kg/m^2^)	21 (45.7)	111 (34.8)	
Overweight (24.5–29.9 kg/m^2^)	12 (26.1)	92 (28.8)	
Obese (>30 kg/m^2^)	9 (19.5)	71 (22.3)	

Smoking			
Never smoker, *n* (%)	25 (54.4)	180 (56.4)	0.598
Former smoker, *n* (%)	10 (21.8)	51 (16.0)	
Current smoker, *n* (%)	11 (23.8)	88 (27.6)	

Smoking amount (pack-year), *n* (%)			
0	25 (54.4)	180 (56.4)	0.868
1–10	7 (15.2)	53 (16.6)	
11–20	7 (15.2)	51 (16.0)	
>20	7 (15.2)	35 (11.0)	

TB history, *n* (%)			
Yes	4 (8.7)	8 (2.5)	0.051
No	42 (91.3)	311 (97.5)	

TB subgroup, *n* (%)			
Lung TB	2 (50.0)	3 (37.5)	0.894
Extrapulmonary TB	1 (25.0)	3 (37.5)	
Unknown	1 (25.0)	2 (25.0)	

Chronic disease *n* (%)			
HT	7 (15.2)	33 (10.3)	0.323
DM	4 (8.7)	28 (8.8)	0.985
Rheumatologic disease	23 (50.0)	151 (47.3)	0.735
Coronary artery disease	4 (8.7)	19 (6.0)	0.512
Obstructive lung diseases	2 (4.3)	**52 (16.3)**	**0.033** ^*∗*^
MS	4 (8.7)	13 (4.1)	0.249
Malignancy	3 (6.5)	12 (3.8)	0.417
Hypothyroidism	3 (6.5)	13 (4.1)	0.437
Cardiac arrhythmia	0 (0.0)	7 (2.2)	0.603
IBH	3 (6.5)	32 (10.0)	0.597
CRF	2 (4.3)	9 (2.8)	0.636

Drugs used, *n* (%)			
Biological treatments	20 (43.6)	149 (46.7)	0.681
Corticosteroid	18 (39.1)	112 (35.1)	0.594

COVID-19 vaccine status, *n* (%)			
Done	4 (8.7)	46 (14.4)	0.291
Not done	42 (91.3)	273 (85.6)	

CRF: chronic renal failure; DM: diabetes mellitus; HT: hypertension; IBD: inflammatory bowel disease; MS: multiple sclerosis; TBF: STANDARD ETB-Feron ELISA (TBF) test. ^*∗*^*p* value < 0.05 was considered statistically significant. Values below 0.05 are in bold and indicate statistically significant differences between two categories.

## Data Availability

The data used to support the findings of this study are available from the corresponding author upon request.
